# Correction: The lithic assemblages of Donggutuo, Nihewan basin: Knapping skills of Early Pleistocene hominins in North China

**DOI:** 10.1371/journal.pone.0189565

**Published:** 2017-12-07

**Authors:** Shi-Xia Yang, Michael D. Petraglia, Ya-Mei Hou, Jian-Ping Yue, Cheng-Long Deng, Ri-Xiang Zhu

The following information is missing from the Funding section: This work was supported by Chinese Academy of Sciences grant XDPB05 to SXY and YMH.

The first email address listed for Shi-Xia Yang is incorrect. The correct address is yangshixia@ivpp.ac.cn.
