# Supplementary material for: Correction: The lithic assemblages of Donggutuo, Nihewan basin: Knapping skills of Early Pleistocene hominins in North China
Source: PLoS One. 2017 Oct 19;12(10):e0186995. doi: 10.1371/journal.pone.0186995 (PMC5648244; doi:10.1371/journal.pone.0186995)
Supplement: S1 File — (PDF) [file pone.0186995.s001.pdf]

PLOS | ONE

RESEARCH ARTICLE

The lithic assemblages of Donggutuo, Nihewan basin: Knapping skills of early pleistocene hominins in North China

Shi-Xia Yang[1,2]*, Michael D. Petraglia[3], Ya-Mei Hou[2], Jian-Ping Yue[2,4], Cheng-Long Deng[1,4], Ri-Xiang Zhu[1,4]

1 State Key Laboratory of Lithospheric Evolution, Institute of Geology and Geophysics, Chinese Academy of Sciences, Beijing, China, **2** Key Laboratory of Vertebrate Evolution and Human Origins of Chinese Academy of Sciences, Institute of Vertebrate Paleontology and Paleoanthropology, Chinese Academy of Sciences, Beijing, China, **3** Department of Archaeology, Max Planck Institute for the Science of Human History, Jena, Germany, **4** University of Chinese Academy of Sciences, Beijing, China

* sxyang@ivpp.ac.cn, yangshixia122@gmail.com

Check for updates

OPEN ACCESS

Citation: Yang S-X, Petraglia MD, Hou Y-M, Yue J-P, Deng C-L, Zhu R-X (2017) The lithic assemblages of Donggutuo, Nihewan basin: Knapping skills of early pleistocene hominins in North China. PLoS ONE 12(9): e0185101. https://doi.org/10.1371/journal.pone.0185101

Editor: William Oki Wong, Institute of Botany, CHINA

Received: June 20, 2017

Accepted: September 6, 2017

Published: September 21, 2017

Copyright: © 2017 Yang et al. This is an open access article distributed under the terms of the Creative Commons Attribution License, which permits unrestricted use, distribution, and reproduction in any medium, provided the original author and source are credited.

Data Availability Statement: All relevant data are within the paper.

Funding: This research is supported by grants from the National Natural Science Foundation of China (Nos. 41602021, 41690112, 41621004 and L1524016; http://www.nsfc.gov.cn/), under the "ideas" to work on early human behaviors in North China. Additional funding for analyses was contributed by Ministry of Science and Technology of China (No. 2014FY110300; http://program.most.gov.cn/). The funders had no role in study design,

Abstract

Donggutuo (DGT) is one of the richest archaeological localities in the Nihewan Basin of North China, thereby providing key information about the technological behaviours of early hominins in eastern Asia. Although DGT has been subject of multiple excavations and technological studies over the past several decades, few detailed studies on the lithic assemblages have been published. Here we summarize and describe the DGT lithic assemblages, examining stone tool reduction methods and technological skills. DGT dates to ca. 1.1 Ma, close to the onset of the mid-Pleistocene climate transition (MPT), indicating that occupations at DGT coincided with increased environmental instability. During this time interval, the DGT knappers began to apply innovative flaking methods, using free hand hard hammer percussion (FHHP) to manufacture pre-determined core shapes, small flakes and finely retouched tools, while occasionally using the bipolar technique, in contrast to the earlier and nearby Nihewan site of Xiaochangliang (XCL). Evidence for some degree of planning and predetermination in lithic reduction at DGT parallels technological developments in African Oldowan sites, suggesting that innovations in early industries may be situational, sometimes corresponding with adaptations to changes in environments and local conditions.

Introduction

In assessments of stone tool assemblages of Eastern Asia, archaeologists have frequently held that there are long periods of stasis, with no significant technological changes until the upper part of Late Pleistocene [1–3]. Yet, investigators working in Eastern Asia continue to lack a detailed knowledge about Pleistocene lithic assemblages in the region, and there are substantial geographic and temporal gaps in our understanding of the archaeological record across this vast area. Though it could be argued that stone tool technologies may have remained relatively conservative over long periods, it is difficult to imagine that hominins never altered or modified their stone-tool using behaviours in the face of unstable and changing climates in northern

data collection and analysis, decision to publish, or preparation of the manuscript.

Competing interests: The authors have declared that no competing interests exist.

latitudes during the Early and Middle Pleistocene. Paralleling the situation in Eastern Asia, early lithic industries with core-flake production have been typically categorised under holistic classifications, such as Oldowan or Mode 1 [4, 5]. Some stone tool analysts, however, have pointed out that early lithic assemblages, usually grouped as simple core-flake industries, sometimes show substantial variability in their flaking and production strategies in African and Eurasian contexts [6, 7]. In fact, detailed lithic analyses and refitting studies on the Lokalalei 2C assemblages in Kenya, showed that Late Pliocene knappers practiced considerable foresight in raw material procurement and lithic manufacture [8]. At the same time, the Lokalalei investigations indicated significant inter-site differences in Late Pliocene and Early Pleistocene sites across Eastern Africa, thereby deconstructing the notion that the Oldowan itself was a homogeneous and static entity over evolutionary time. Likewise, examination of the lithic assemblages at Omo (Member F) indicated that, despite the limitations of the small quartz clasts, early hominins knapped cores in a precise and systematic fashion, suggesting deliberate and rational methods in obtaining flakes [9].

Such observations suggest the need to re-evaluate technological trends in Early Pleistocene lithic industries of eastern Asia, which are often regarded as monotonous and unchanging.

The Nihewan Basin, with a series of early sites dated between the Gauss–Matuyama and Matuyama–Brunhes geomagnetic reversals (2.58–0.78 Ma), is an ideal region to examine Early Pleistocene archaeological sites and technological trends in eastern Asia. The DGT site, first identified and investigated in 1981, contains thousands of lithic artefacts, representing one of the richest localities in the Nihewan Basin. Although multiple excavations and lithic assemblage studies have been conducted at DGT [10–14], there has been little agreement among researchers about the behavioural importance of the site, in part owing to the lack of a detailed and comprehensive analysis of the lithic assemblages and its place relative to other Nihewan sites. To rectify this situation, here we analyse the lithic assemblages of DGT, providing an opportunity to reassess stone tool knapping methods. Moreover, given that technological information from Xiaochangliang (XCL) was recently collected [15], inter-site trends in lithic reduction between two Early Pleistocene sites of the Nihewan Basin are now possible.

Site setting and study history

The DGT site, situated in the eastern margin of the Nihewan Basin (40˚13'22"N, 114˚40'11"E, Fig 1), is considered one of the most important Palaeolithic sites in China [16–18]. The Nihewan Basin is a large fault-related basin, composed of the Yuxian and Yangyuan Basins (Fig 1A–1C). The basin measures ca. 150 to 200 km^2, and is cross-cut by the Sanggan River, which meanders across the area [19]. The basin is filled with Late Pliocene to Holocene lacustrine, fluvial and aeolian deposits (in recent years the term "Nihewan Formation" has been used to define the whole fluvio-lacustrine sequence in the basin). The DGT section is ca. 44.8 m in thickness, and the main part consists of the Nihewan Beds with a thickness of about 37.4 m, capped by the last glacial loess (4.5 m) and soil associated with the last interglacial (2.9 m) and underlain by Jurassic breccia (Fig 1B, [20, 21]). The main cultural layer described here is located in the lower part of the section, ranging over a thickness of 6.5 m.

DGT was first identified and excavated in 1981 [10]. After its initial investigation, several excavation seasons were sponsored by the Institute of Vertebrate Paleontology and Paleoanthropology (IVPP) of the Chinese Academy of Sciences. A joint Sino-U.S. excavation was conducted in 1991–1992, and later, between 1997 to 2001, several small-scale excavations were conducted by the IVPP (Table 1; [22]). Thousands of lithic artefacts and mammal fossils were collected from the excavations, and the DGT site rose to prominence, as it provided new information about early human behaviours in China [14, 23].

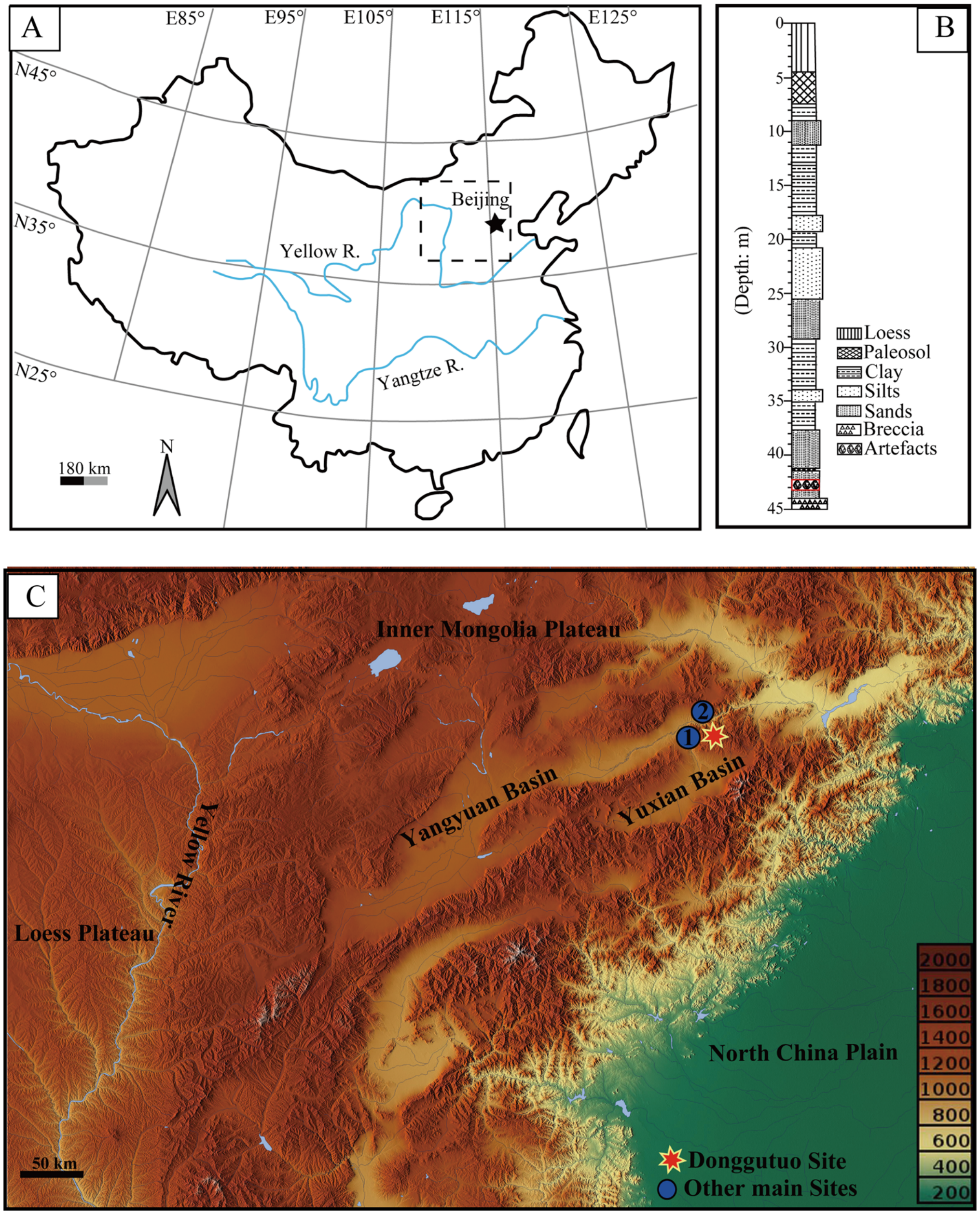

Fig 1. DGT, Nihewan Basin, China. (A) The Nihewan Basin, showing the location of Early Pleistocene sites; (B) The general stratigraphy of DGT, showing the location of the main artefact horizon; (C) The location of DGT and other key sites (1. Xiaochangliang, 2. Cenjiawan).

https://doi.org/10.1371/journal.pone.0185101.g001

The cultural layers of DGT ranged over a thickness of 6.5 m, though the main concentration of lithic artefacts and fossils were in the middle part of the section, at a depth interval ranging between 41.6 to 44.8 m. According to the 2000–2001 excavations, more than 96% of the artefacts were from the middle part of the section [24]. Most of the recovered mammal fossils were small fragments, without heavy weathering [25], though few fossils could be identified to species level. Nevertheless, some vertebrate fossils from the cultural deposits could be identified as *Myospalax. fontanieri*, *Canis* sp., *Palaeoloxodon* sp., *Equus. sanmeniensis*, *Coelodonta. antiquitatis*, *Bison* sp., and *Gazella* sp. [10]. Preliminary faunal analyses indicated that carnivore-

Table 1. Excavation field seasons at DGT.

Field season	Excavation team	Excavated area	No. of artefacts	Key publications
1981–1983	IVPP	45m^2	1443–1676[a]	[10–12, 14, 27]
1991–1992	Sino-U.S. joint team	30 m^2	?	none
1997	IVPP	12 m^2	702	[12, 26]
2000–2001	IVPP	12m^2	974	[25]

[a]1443 is the number given in the 1985 publication. Later studies reported different total numbers of lithic artefacts (i.e., [11]: 1432 pieces; [12, 27]: 1571 pieces; [14] 1676 pieces).

https://doi.org/10.1371/journal.pone.0185101.t001

gnawed bone and cut-marked bone each formed 1.2% of the fossil assemblage [25]. Lithic refits, the presence of small lithic shatter (<25 mm), and fresh artefact edges indicated that the archaeological materials did not experience significant postdepositional disturbances, but accumulated over time [14].

One of the key debates to emerge from the various studies of the lithic industries was the degree to which knappers controlled their flaking and the degree to which the lithic products were planned at the outset. The original investigators working on DGT described the use of both hard hammer percussion and the bipolar method to obtain flakes, noting the presence of some finely retouched flakes [10]. Later studies indicated that the lithic assemblages were dominated by flakes and flake fragments from casual cores, with the use of irregular preparatory core reduction methods [11].

Hou [26] first noted and described the presence of small, prepared cores at DGT, naming them as the "Donggutuo Shaped core". Subsequently, Hou named this distinctive flaking method as the "Donggutuo core", describing the prepared cores as wedge-shaped in order to produce small elongated flakes [12, 23, 27]. However, others were skeptical of this claim, and argued that the so-called prepared cores from DGT were simply a variant of standard cores [16, 28] or even a product of bipolar reduction methods [14]. Hence, although the lithic assemblages of DGT have been remarked upon by a number of analysts, there is little agreement about stone tool reduction methods, and in particular whether prepared core techniques and particular flaking products are in fact present. This situation appears to have arisen, in part, as a consequence of limited first-hand studies and the lack of a comprehensive analysis of the lithic assemblages from the multiple excavations.

Chronology and environmental background

Magnetostratigraphic dating of the DGT cultural layers has been conducted by several scholars since the 1980s' [20, 29–31]. According to the most recent magnetostratigraphic research [20, 31], the age of the DGT cultural layer is just prior to the onset of the Jaramillo normal subchron, which has been dated at 1.053±0.006 Ma [32] or 1.072 Ma [33]. Wang and colleagues [20] indicated that the short interval of possible geomagnetic excursion (E3) within the pre-Jaramillo Matuyama reverse chron (encompassed within the DGT artefact layer) may be correlated to the Punaruu geomagnetic excursion, which has a $^{40}Ar/^{39}Ar$ age determination of 1.105 ±0.005 Ma [32]. This lends further support to the contention that the DGT cultural layers date to ca. 1.1 Ma [20]. Subsequently, Li et al. [31] claimed that the DGT artefact layers occur around the Cobb Mountain geomagnetic excursion based on investigations of magnetostratigraphy and sediment grain size, estimating its age to be 1.204–1.119 Ma. Considering the magnetochronological data together, we estimate the age of DGT as 1.1–1.2 Ma. This age is close to the onset of the mid-Pleistocene climate transition (MPT), which began ca. 1.25 Ma [34] or ca. 1–0.8 Ma [35–37]. Based on the magnetostratigraphic data from the Nihewan Basin in general,

the age of the DGT site is younger than that of other key archaeological sites, such as Majuangou, Xiaochangliang and Banshan [17, 38, 39].

A number of palaeoenvironmental studies were conducted on the Nihewan Formation and on the archaeological sites within the sequence [24, 40–42], including a multidisciplinary investigation on the DGT section [24]. Investigators have reported that most of the lithic artefacts (88%) were from Stage II, a deposit characterized grey to greyish-yellow silt. The pollen analysis of the Stage II horizon indicated the presence of a temperate forest and humid forest grass steppe condition [24]. Analysis of iron oxides estimated temperature to be 7.28˚C lower than the present average annual temperature of 7.5˚C, indicating much colder conditions [24].

Today, the Nihewan Basin is located at the northeastern edge of the Loess Plateau. DGT roughly corresponds to the transition between the Wucheng and Lishi Formations of the loess/palaeosol sequence [36]. Sediment grain size, rock magnetism and the pollen analyses of these Formations [24, 43–46], indicated significant environmental changes and fluctuations in North China, e.g., increased aridification in high-latitude areas, stepwise southerly migrations of the Mu Us desert lying to the north of the Chinese Loess Plateau, and C4 plant expansions in the Loess Plateau region.

Climate records indicated changes in the length and intensity of the glacial-interglacial cycles, with the dominant periodicity of high-latitude climate oscillations changing from 41 kyr to 100 kyr [34, 35, 37, 47]. This variability was accompanied by a series of global or regional palaeoclimatic and palaeoenvironmental changes, such as the increase in aridity and monsoonal intensity in Asia and Africa and decreases in sea surface temperatures in the North Atlantic and tropical-ocean upwelling regions [34]. The DGT occupations therefore correspond with a changing and unstable environment.

Results

Raw material selection

The great majority of raw materials used by the DGT knappers was chert, forming 96% (n = 2315) of the lithic assemblage examined here. The remaining 4% of the artefacts were made on quartz, volcanic breccia and andesite. Previous investigators working on DGT indicated that the main raw materials were likely from the chert breccias in the Jurassic pyroclastic rocks, 200–600 m from the DGT site [48]. The fractures formed by tectonic movement in the chert breccias resulted in the production of small asymmetric and sub-angular nodules useful for lithic reduction [48]. The available clasts from the breccia were of various qualities, including fine-grained pieces and those which had internal flaws with retention of a significant amount of interstitial material. Hominins heavily exploited the chert breccias at both DGT and XCL, as both sites were within 1000 m of each other, and located close to the same raw material source [15].

DGT lithic assemblage

In the current study, a total of 2315 lithic artefacts, stored in the IVPP, and recovered from the field seasons conducted in the 1980s and in 1997 were analyzed, forming the majority of the previously published information. Table 1 is a compilation of excavation data by field season, including reports of the total number of lithic artefacts retrieved [11, 14, 27]. Unfortunately, the 1991–92 field data were not published, and the artefacts could not be located at the time of our analysis; moreover, the 974 lithic artefacts retrieved in the 2000–01 season were not available for our study.

As indicated in Table 1, freehand hard hammer percussion (FHHP) is the predominant flaking method as illustrated in the cores, flakes and flake fragments across all studies. Here we

identify 558 pieces of shatter, though this class of material was not recorded in previous studies (Fig 2). Pieces of shatter are usually smaller than 25 mm, showing no signs of conchoidal percussion features on upper or lower faces. In previous publications, the number of flakes is much larger than the current study; for example, in the 2014 report [14], 924 flakes were identified, likely indicating that some of the broken flakes or splinters were classified as flakes.

In the original publication of DGT, Wei [10] reported the presence of bipolar products, and later independently confirmed by Shen and colleagues [49]. Here we confirm that bipolar cores and splinters are present, as shown by the presence of double bulbs of percussion on the splinters or the flaked pieces and battering scars on two flaked edges (Fig 3). Though bipolar cores and splinters are less frequent in comparison to FHHP products (Table 2), bipolar percussion still forms a key reduction method at DGT.

The proportion of retouched pieces across various studies is generally high, forming ca. 10% of the lithic assemblage (Table 2) (NB: the 1991 report distinguished modified pieces from retouched pieces, but all later studies classed these as retouched pieces or tools). The high percentage of retouched pieces are an important characteristic of the DGT assemblage, which will be described in more detail below.

Knapping methods

Freehand hard hammer percussion and bipolar methods identified at DGT are described below. Though recent studies show that the two methods are sometimes difficult to distinguish, a combination of qualitative and quantitative methods is considered the best approach for their categorization [50–53].

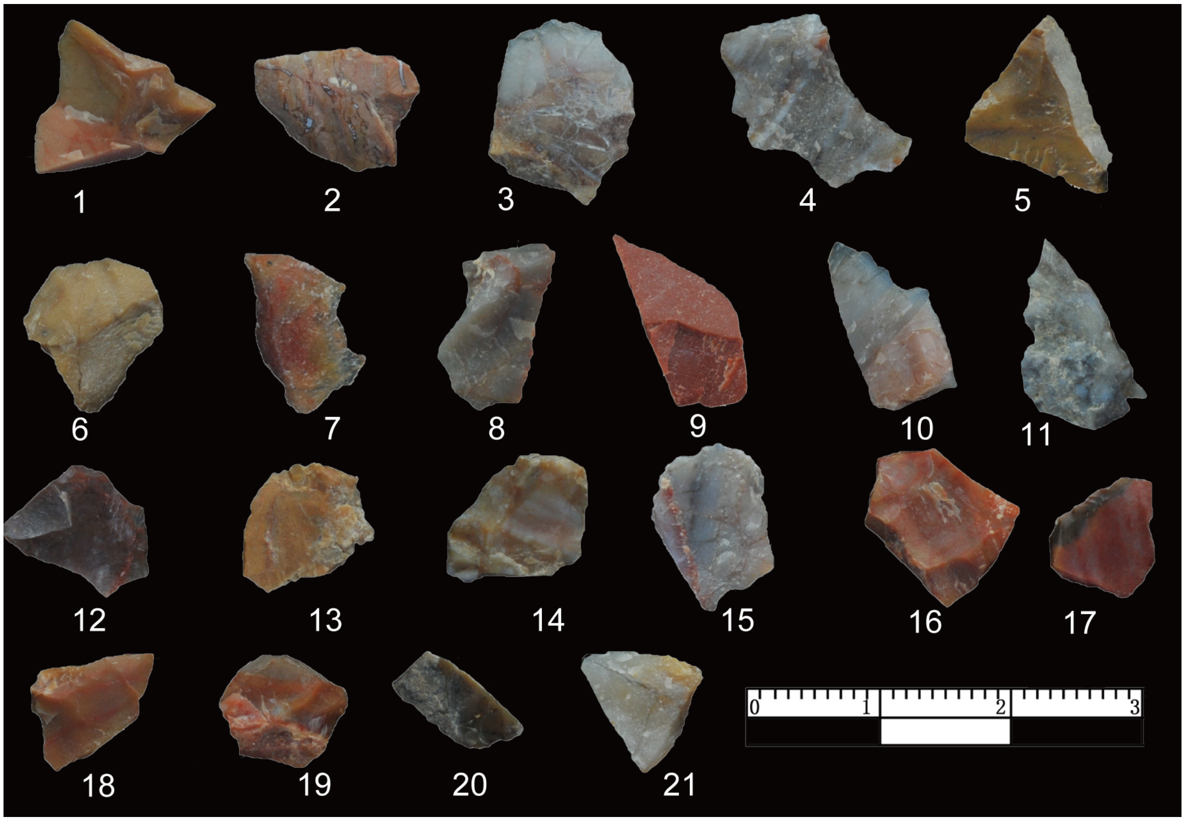

Fig 2. Shatter from DGT. Shatter is typically smaller than 25 mm. Shatter have no clear sign of conchoidal percussion and it is often difficult to distinguish upper and lower faces.

https://doi.org/10.1371/journal.pone.0185101.g002

Freehand reduction

DGT had a marked increase in FHHP products (i.e, cores, flakes, flake fragments), accounting for 77.47% of the reduction system, in comparison to the lower percentage found at XCL (43.06%, see [15]). A total of 245 cores, 380 flakes and 300 flake fragments and splinters are identified as the product of freehand percussion methods (Table 2). Raw materials used for artefact production include chert, volcanic breccia and quartz, though chert predominates, representing 97.73% (n = 904) of the total FHHP assemblage.

Cores and the developed core-flake technique

The cores have an average maximum length of 37.8 mm and only four exceed 100 mm (Table 3). Based on the number and morphology of platforms, FHHP cores were sub-divided

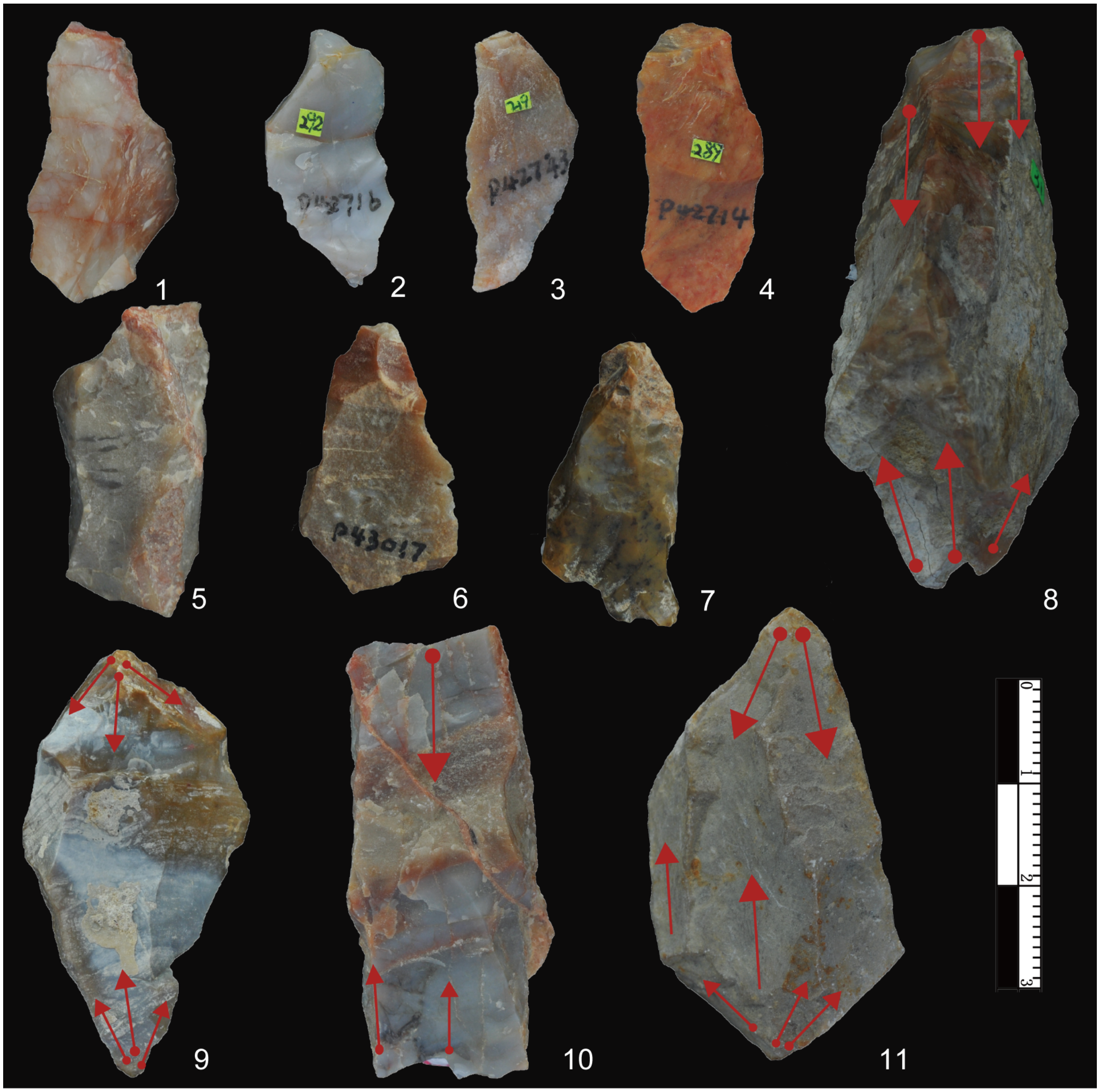

Fig 3. Bipolar products from DGT. No. 1–7 are splinters from bipolar percussion; No. 8–11 are bipolar cores which show percussion or battering scars on two edges (the arrows indicate the opposed flaking scars).

https://doi.org/10.1371/journal.pone.0185101.g003

Table 2. Lithic classifications of DGT artefacts according to previous studies and the current study.

Lithic class	1991[a]	%	2000[b]	%	2014[c]	%	Current Study[d]	%
Core (Freehand)	66	4.61	142	9.04	147	8.77	245	10.58
Core (Bipolar)	—	—	—	—	4	0.24	65	2.81
Flake (Freehand)	888	62.01	364	23.17	920	54.89	380	16.41
Flake fragment		—	—	—	—	—	300	12.96
Splinter (Bipolar)	—	—	—	—	5	0.3	204	8.81
Modified pieces	143	9.99	—	—	—	—	—	—
Retouched pieces	10	0.70	165	10.50	230	13.73	228	9.85
Utilized flakes	41	2.86	—	—	—	—	—	—
Shatter	—	—	—	—	—	—	558	24.11
Angular fragment	284	29.40	900	57.27	370	22.07	335	14.47
TOTAL	1432	100	1571	100	1676	100	2315	100

[a] [11]

[b] [27]

[c] [14]: all three studies are based upon the artefacts recovered in 1981; in each, the numbers vary slightly.

[d] The current study comprises 2315 lithic artefacts recovered from the 1981 and 1997 excavations.

https://doi.org/10.1371/journal.pone.0185101.t002

into five main types (i.e., Unidirectional, Bidirectional, Multidirectional, Bifacial, Wedge-shaped) (Figs 4 and 5).

Unidirectional cores exploited from a single flaking direction were the most common type (n = 133, 70.37%). The unidirectional method was a simple flaking technique, and most cores show natural or plain platforms (Fig 4, no. 3). Retouched platforms were exhibited on a small number of unidirectional cores (Fig 4, no. 1, n = 14, 13.59%), and retouch scars were clearly visible on the platforms. The Bidirectional cores were flaked from two opposite platforms, though with no platform preparation (n = 15, 7.94% Fig 4, no. 3). The third type are Multidirectional cores (n = 21, 11.11%, Fig 4, no. 2), which were often irregular in flaking patterns, with removals showing no clear organization of the reduction process. The fourth type, Bifacial cores, are alternately knapped along edges (n = 11, 5.82%, Fig 4, nos. 4, 5), showing that the knappers used former flake scars as platforms to produce the follow-on flake.

The fifth type of core, the Wedge-shaped core, was previously described by Hou [26], and later named as the "Donggutuo Core" [12, 23, 27]. A total of 9 Wedge-shaped cores were identified in this study, forming 3.67% of the core assemblage (Fig 5). The average maximum length is of 26.7 mm, and average maximum width is 43.1 mm. The average platform thickness is 25.9 mm. Three of the nine cores have prepared platforms, and the 6 others have plain platforms. The Wedge-shaped cores typically have a single platform from which flakes were struck. The number of visible negative flake scars on each of the core flaking surfaces range between 4 to 6. This wedge-shaped flaking method produced predictably small and elongated flakes, which are often micro-blade-like in form (Fig 6, nos. 12, 13).

Table 3. DGT lithic types by number and size (mm), subdivided by reduction technique.

Technological System	Main categories	No.	%	Length		Width		Thickness	
				Mean	Std.D	Mean	Std.D	Mean	Std.D
Freehand	Core	245	20.52	37.80	16.43	49.53	20.29	36.18	19.97
	Flake	380	31.83	26.69	11.1	26.29	11.12	8.90	4.22
	Flake frag.and splinter	300	25.12	31.61	10.72	26.74	10.07	11.09	4.47
Bipolar	Core	65	5.44	36.24	10.68	26.35	9.66	17.22	5.29
	Splinter	204	17.09	30.25	8.43	19.70	6.82	9.94	3.22

https://doi.org/10.1371/journal.pone.0185101.t003

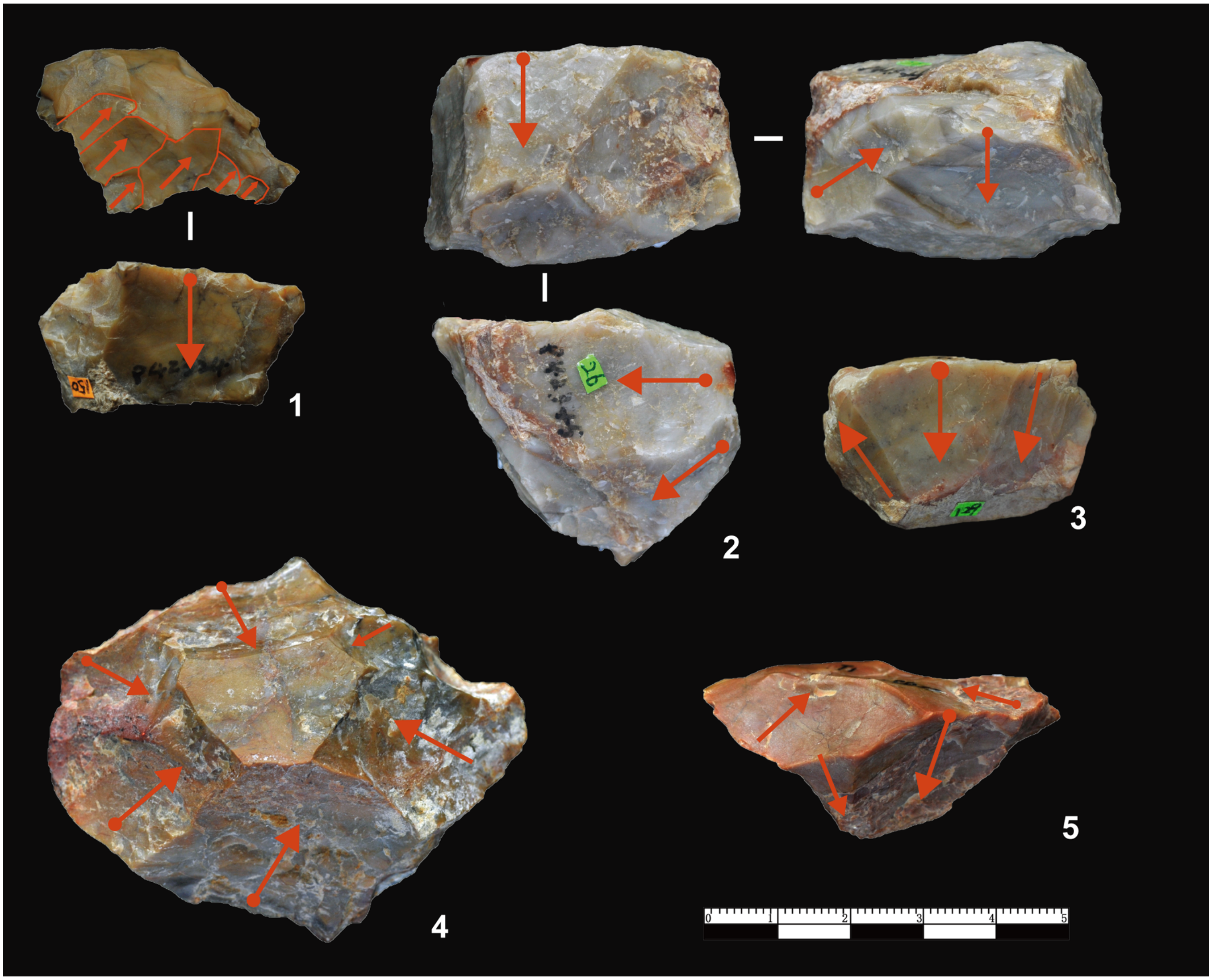

Fig 4. Core types identified at DGT. No. 1 is a Unidirectional core with a faceted platform, showing a series of negative flake scars on a single edge; No. 2 is a Multidirectional core; No. 3 is a bipolar exploited core with two opposed flaking directions; Nos. 4 and 5 are bifacial cores showing alternate flaking patterns.

https://doi.org/10.1371/journal.pone.0185101.g004

Flakes

The flakes have an average maximum length of 26.69 mm (Table 3, Fig 6). Partitioning flakes by their maximum length indicates that 34.4% average less than 20 mm and 60.0% have a maximum length between 21–40 mm. The platform angles on flakes are generally steep and range between ca. 70–90˚, with a cluster around between 80–85˚ (78%). The flake butts can be divided into four main types: plain (n = 94, 59.49%), natural (n = 40, 25.32%), facetted (n = 6, 3.8%) and linear (n = 18, 11.39%).

The DGT flakes have a high percentage of flakes without cortex (30.63%) with high negative flake scar counts, 27.4% of flakes with more than 3 dorsal negative scars (Fig 7). The increase of the retouched platforms and the negative flake scars indicates the development of the capacity of core exploitation. These quantitative results reinforce previous observations which suggested the presence of cores with platform preparation and a more complicated exploitation system.

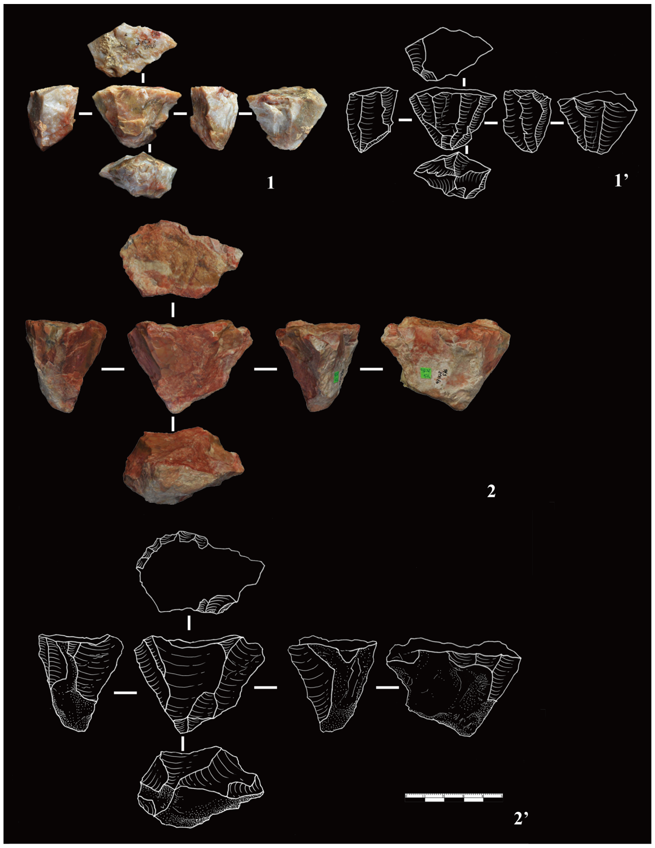

Fig 5. Wedge-shaped cores from DGT. Wedge-shaped cores were previously described and named by Hou [12, 23, 26, 27]. The cores have single platforms which are sometimes prepared, resulting in the striking and production of a series of small, enlongated flakes. No.1 is a heavily exploited core, with negative flakes scars visible on every face. No. 2 is partly exploited, though showing platform preparation and the striking of small flakes from a single platform. The two examples are similar show the reduction direction and the morphology of the "DGT core".

https://doi.org/10.1371/journal.pone.0185101.g005

A total of 300 flake fragments and splinters were also identified as FHHP byproducts, representing a relatively high proportion of the lithic assemblage, comprising 12.96%. The broken flakes include "siret" flakes [55], i.e., flakes with broken platforms or without platforms. Faced with the similar sizes of the chert nodules, the DGT habitants preferred the FHHP method. FHHP provided regular flakes and platform preparation allowed knappers to work irregular clasts of chert in a more controlled fashion.

Bipolar reduction

Although FHHP products were frequent at DGT, bipolar reduction was an important method, accounting for 22.53% of the lithic assemblage (Table 3). A total of 65 bipolar cores and 204 splinters were identified (Fig 3). Bipolar splinters are defined here as small pieces with crushing on either the platform or the base, and always without evidence of Hertzian initiation (e.g., bulbs of force, eraillure scars, ripple marks) [56].

The bipolar cores have an average maximum length of 36.24 mm, slightly smaller than that of freehand cores (Table 3). The bipolar cores often had a stable relationship between the platform and the base, with striking typically from a single direction. The bipolar splinters have an average maximum length of 30.25 mm (Table 2), thus somewhat larger than whole freehand flakes.

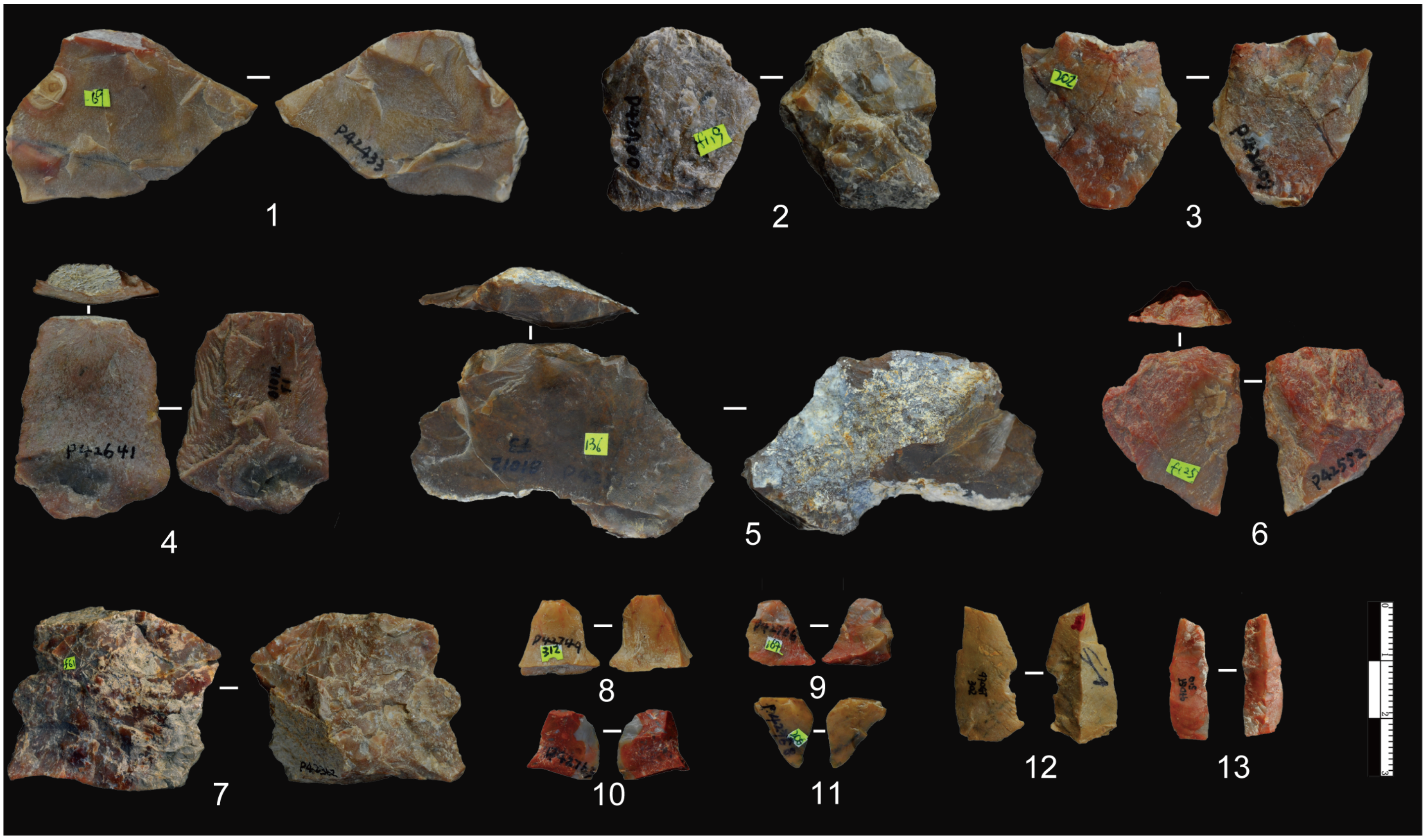

Fig 6. Freehand flakes from DGT. The freehand flakes show Hertzian initiation, i.e. waves, bulbs of force, distinct striking platforms.

https://doi.org/10.1371/journal.pone.0185101.g006

Retouched pieces

A total of 228 retouched artefacts were identified at DGT (Figs 8, 9 and 10), accounting for 9.85% of the lithic assemblage. The retouched pieces are generally small in size with an average maximum length of 31.88 mm, and more than 50% are smaller than 30 mm, and 14% are

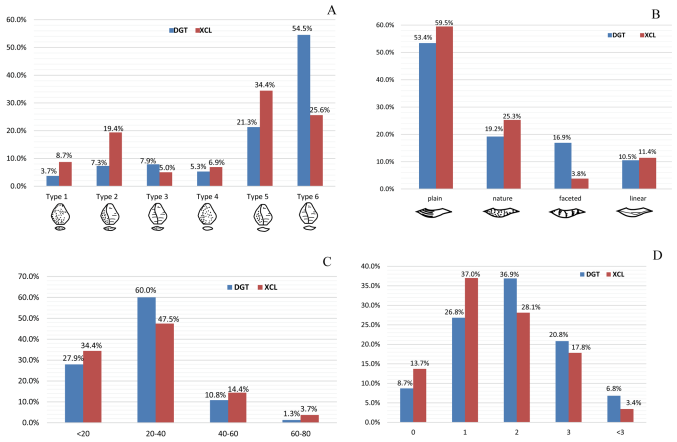

Fig 7. Comparison of DGT and XCL flake attributes. (A) Percentage of cortex on flakes according to Toth's types [54]; (B) Types of striking platforms on flakes; (C) Flake size ranges (in mm); (D) Number of negative scars on dorsal faces of flakes.

https://doi.org/10.1371/journal.pone.0185101.g007

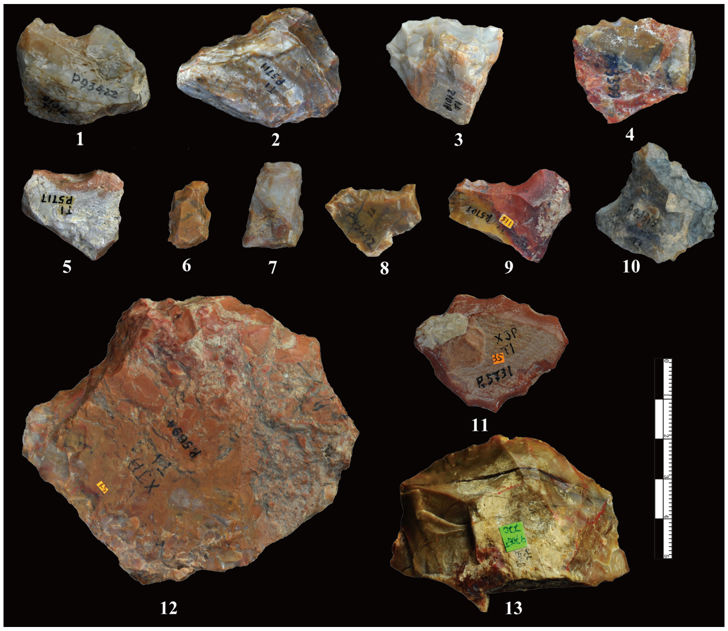

Fig 8. Retouched pieces from DGT. No. 1, 5: notches; No. 2, 8, 9, 12, 13: Scrapers with continuous retouch along edges; No. 3, 4: denticulates showing uneven edges with more than three retouch scars; No. 6, 7, 10, 11: pieces with retouch on multiple edges.

https://doi.org/10.1371/journal.pone.0185101.g008

smaller than 20 mm. The repeated location of the retouch on the same portion of the blanks (Figs 9 and 10), their invasive depth as well as the freshness of the adjacent edges strongly suggest intentional manufacture. The average maximum retouch extent was 32.07 mm, and sometimes exceeding the maximum length of the pieces, as several margins were retouched including on a convex edge (Fig 8, nos. 2, 13), on two to three edges, and occasionally on every edge (Fig 8, nos. 7, 11).

The DGT retouched pieces can be typed as scrapers, denticulates, notches, borers and points. The large majority are scrapers (78%), which include straight, concave and convex edges. A relatively high percentage of scrapers (24%) are with abrupt retouched angles (>60˚). The notches and denticulates (Fig 8, nos. 1, 3, 4, 6) can also be considered general tool forms. Most of the notches are made by one retouch scar, whereas there are 3 pieces with more complex notches, made by applying several retouch removals. The average maximum length of the notches is 30.06 mm, and the average depth of the notches is 7.47 mm. The denticulates can be readily distinguished from scrapers, as they are with continuous small notches on one edge (e.g., Fig 8, no. 4). The denticulates are somewhat larger than other retouched pieces, with an average maximum length of 36.14 mm. In addition to these general tool types, borers with small tips and point like tools were identified (Fig 9). Borers were retouched in order to form a small tip (Fig 9, nos. 2–4), the average of tips measuring 7.94 mm. Only three pointed tools were identified, the pointed tools have clear bifacial retouch on two edges, with a triangular profile (Fig 9, no. 1). Some retouched pieces (Fig 8, no. 6) are difficult to type (i.e., unidentified retouched pieces), and they are typically small in size and often show irregular retouch.

Comparisons between DGT and XCL

The DGT and XCL sites are located within 1000 m to each other, though DGT is about 160–260 kyr younger in age. Though the hominins at each site predominantly used the same chert

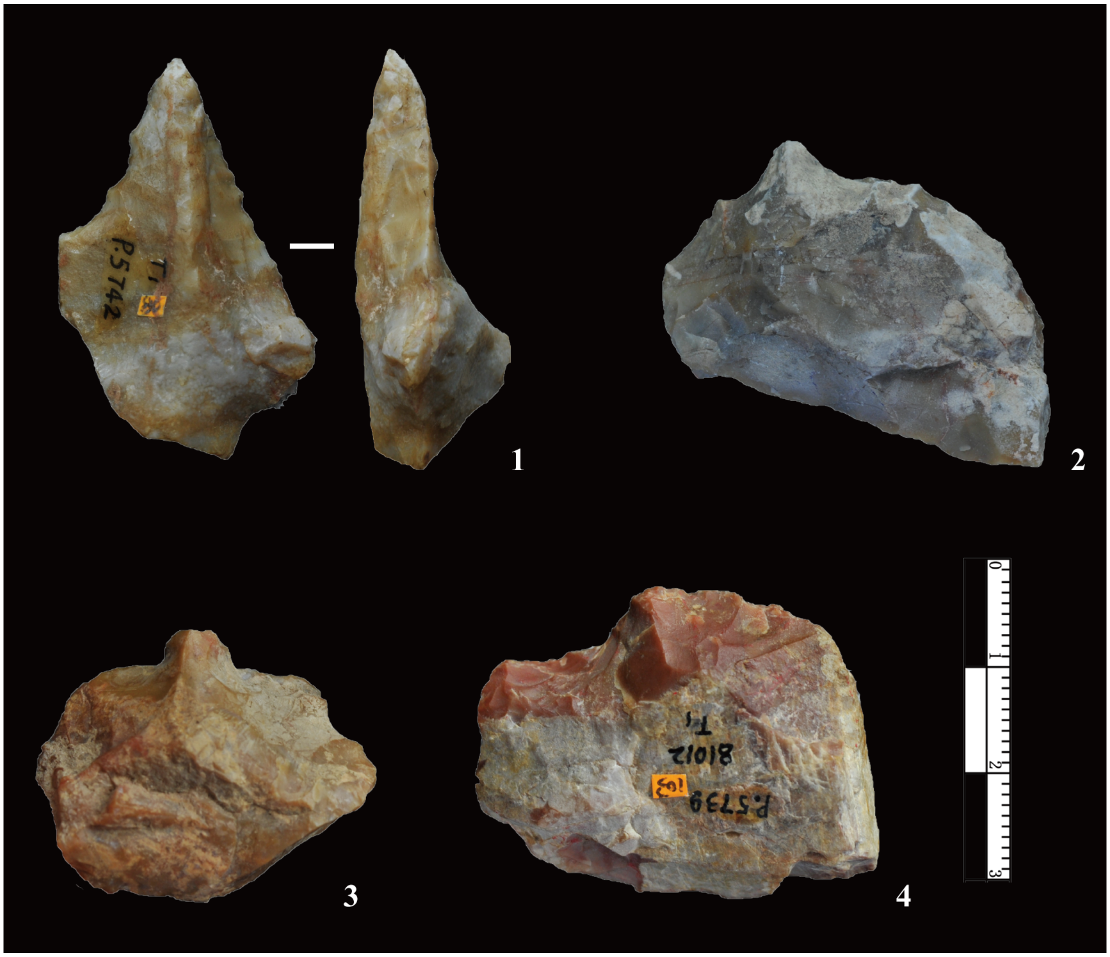

Fig 9. Point and borers from DGT. No.1: point with retouch on ventral and dorsal faces and along two edges on both sides to form a tip. The retouch is systematic and the length on the two converging edges are 18.7 mm and 31.1 mm respectively; No. 2–4: borers displaying retouch to form short and rounded tips.

https://doi.org/10.1371/journal.pone.0185101.g009

source and materials for lithic production, our comparison indicates some substantive differences in reduction, with DGT knappers applying some innovative flaking methods.

FHHP was clearly the dominant flaking strategy at DGT, forming 77.47% of the assemblage, while at XCL, FHHP comprised about 43.08% [15]. To analyze differences in core and flake production between the two assemblages, we compared flake sizes, core flake size traits and retouched tool sizes. Our comparison indicated that the DGT cores were similar in size with those from XCL, though somewhat shorter in the average maximal length (Table 3, Fig 11A). The DGT flakes were smaller size than the ones from XCL, as more flakes in DGT were in the 20 mm to 40 mm (60.0%) size range (Fig 7C; Fig 11B). DGT showed a significantly higher proportion of faceted platforms in comparison to XCL (Fig 7B). The DGT flakes had a much higher percentage of flakes without cortex (30.63%) (See Types 3 and 6 in Fig 7A), and the negative flake scar counts are increased (Fig 7D). The increase of the retouched platforms and the negative flake scars indicates the development of the capacity of core exploitation. These quantitative results reinforce Hou's previous observations which suggested that cores at DGT have platform preparation and a more complicated exploitation system [27].

XCL had a higher percentage of bipolar cores (14.4%) in comparison to DGT (5.4%). The bipolar cores form XCL were somewhat smaller (<20 mm) (Fig 11C) indicating more

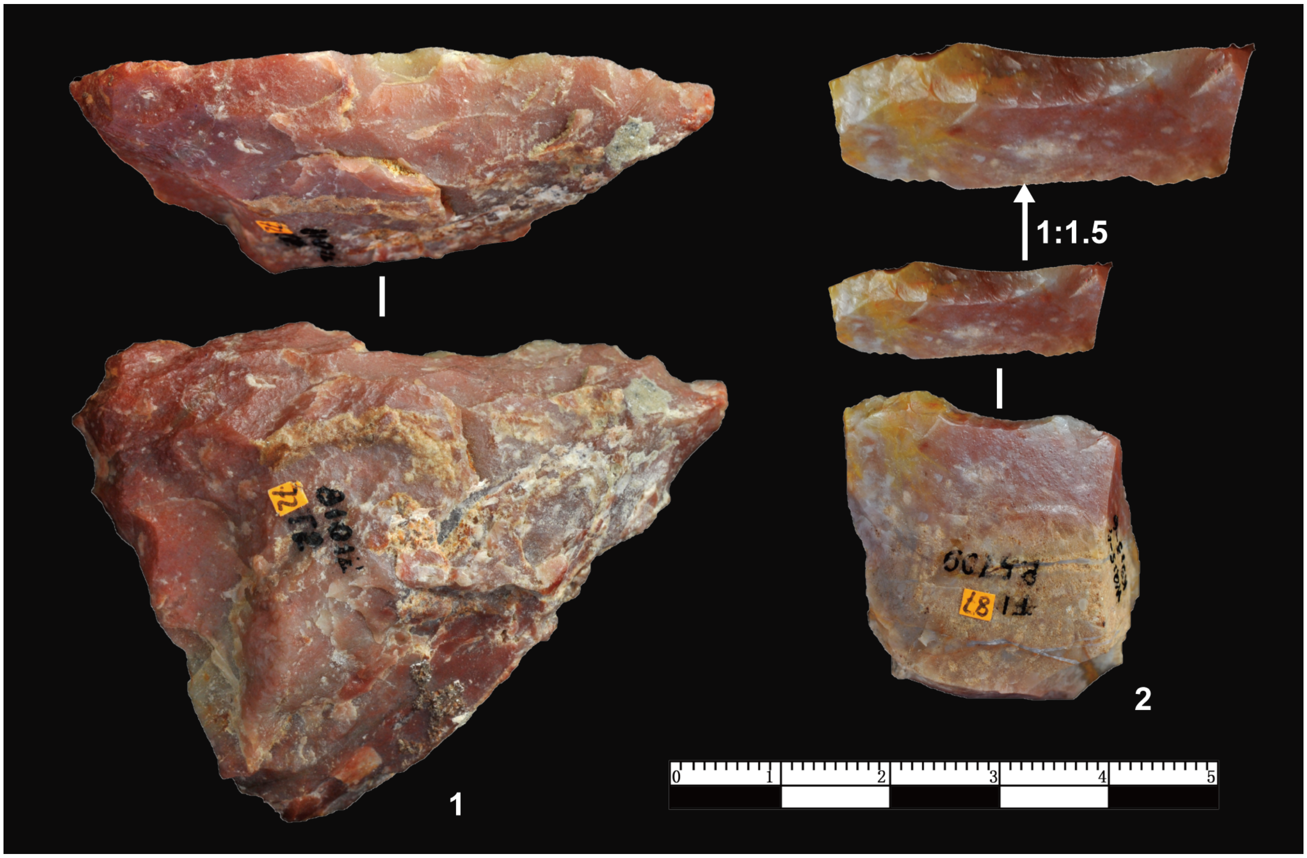

Fig 10. **Profiles of finely retouched pieces from DGT.** No. 1: Repeated retouch along a single working edge (the retouch is inversely applied up to 15 mm).; No. 2: Very fine and regular retouch along a single working edge. The retouched working end is smooth and sharp.

https://doi.org/10.1371/journal.pone.0185101.g010

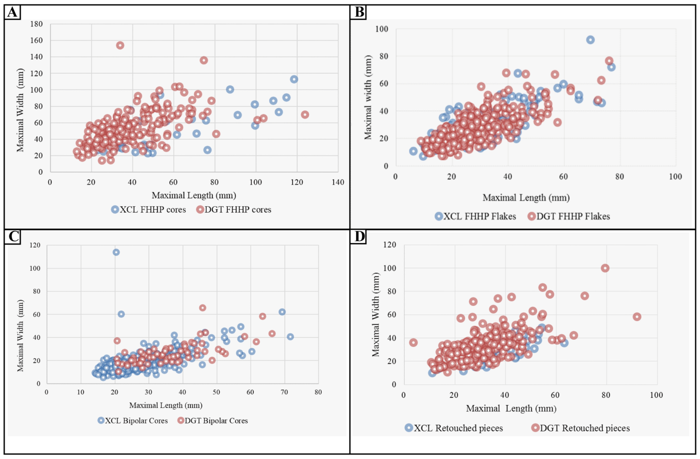

Fig 11. Comparison of stone artefact size distributions from DGT and XCL. (A) Size distribution of bipolar cores from DGT and XCL, indicating that bipolar cores from DGT were larger; (B) Size distribution of FHHP flakes from DGT and XCL, showing that more flakes in DGT were in the 20 mm to 40 mm size range; (C) Size distribution of bipolar cores from DGT and XCL, demonstrating that DGT has more cores smaller than 40 mm; (D) Size distribution of retouched pieces from DGT and XCL, showing that compared to XCL, retouch was on varied sizes of pieces in DGT.

https://doi.org/10.1371/journal.pone.0185101.g011

intensive reduction of clasts. Compared to XCL, the retouched pieces at DGT were much more prominent, the percentage of retouched pieces increasing from 2.95% to 9.85%. The retouch present on pieces varied in size at DGT (Fig 11D). The retouch depth and extent were substantially increased as well, the average maximal length of retouched ends on DGT retouched tools was 31.8 mm whereas at XCL it was smaller (25.86 mm) on average. The retouched tools at DGT show more standardization, and typed here as scrapers, notches, denticulates and borers. The pointed tools at DGT are especially noteworthy in this regard (Fig 9, no. 1), as this type has not been previously recognised in any of the Nihewan Early Pleistocene sites.

In sum, faced with similar sizes of chert nodules, the DGT hominins mostly applied FHHP as opposed to the bipolar technique, used more commonly at XCL. Through FHHP, the DGT knappers were able to produce regular and well controlled flakes. Through platform preparation and the development of wedge-shaped preparatory core methods, the DGT knappers were able to obtain small, slender micro-blades, despite the irregular and small shape of the chert clasts. Flakes produced by such core reduction methods were then shaped into retouched tool types, which were likely used in a range of scraping, cutting and boring tasks.

Discussion

The Nihewan Basin is a remarkable region for understanding the behaviour of early hominins in Eastern Asia given its wealth of archaeological discoveries in stratified and dated contexts. Though a number of excavations have been performed, few lithic assemblages have been examined in any level of detail, with few exceptions [15, 23, 57, 58]. Here we have evaluated the stone tool assemblages from DGT, which has been highlighted as one of the most important sites from the Basin given the large sample of lithics and fossils. Hou's hypothesis [26] that the cores from the site are 'advanced' has formed a debate as to its authenticity [16, 28]. Here we have re-assessed this claim, supporting Hou's contention that the DGT lithic assemblages show innovations in core technology, reduction systems and tool production.

Differences in stone tool technology among Early Pleistocene sites in the Basin are highlighted through comparison of two localities that are in close spatial proximity, i.e., XCL, dating to ca. 1.36 Ma, and DGT, dating to ca. 1.1 Ma. Though hominins at both sites selected and utilized small irregular chert nodules from local sources, the DGT knappers preferentially utilized FHHP as opposed to the more frequent use of bipolar methods at XCL. Core reduction methods at DGT showed some degree of flexibility in the wide range of flaking strategies, including the presence of Wedge-shaped cores (the "DGT Core"). Fig 5 demonstrates that the DGT knappers applied FHHP in a controlled manner, preparing the core platforms with predetermination in order to strike off a series of small flakes, in some cases forming micro-blade-like flakes (Fig 8, no. 12, 13). In addition to both flexible and preparatory core flaking strategies, a key development in the DGT assemblage was the presence of a large number and percentage of retouched pieces in the lithic assemblage. Though retouched pieces were present at XCL, retouched tools are a prominent development at DGT, accounting for nearly 10% of the lithic assemblage (Fig 8). The retouched pieces at DGT show regular application of deep negative scars and extensive flaking along edges, producing long and sharp edges for a variety of tasks. Distinctive tool types were clearly produced by the DGT hominins, and the presence of borers and points is particularly noteworthy as these are rare and unusual tools in Early Pleistocene assemblages (Fig 9).

Given our observations at DGT, it is relevant to note that FHHP was applied on irregular clasts and raw materials at the Nihewan site of Cenjiawan (CJW), also dating to ca. 1.1 Ma [59]. According to published information, and recent observations by one of us (SXY), the

lithic assemblage at CJW is dominated by FHHP, with less frequent bipolar products [58, 60–63], thus similar to DGT. The lithic refitting study at CJW demonstrated multidirectional flaking methods and the continuous rotation of cores and the removal of flakes, with efficient and maximum utilization of the small irregular clasts [58, 62]. Both CJW and DGT therefore appear to show some innovations in flaking methods by hominins in the Nihewan Basin at ca. 1.1 Ma.

The technological innovations at DGT has implications with respect to Early Pleistocene hominin cognition and behaviour. At 1.1 Ma, it appears that hominins were able to adjust their stone tool reduction methods to obtain desirable products from small clasts and poor quality raw materials, something that is evident in earlier lithic assemblages in Africa [9]. The alternate use of FHHP and bipolar methods shows flexibility in their approach to the small and poor quality raw materials. Hominins at DGT were also able to maximize the number and types of flakes from cores, including the application of preparatory techniques on cores to obtain desired flakes. The regular and systematic production of retouched pieces, including some very different tool forms, suggest that hominins produced items for particular activities, such as scraping, cutting and boring.

On the whole, the technological evidence at DGT indicates that Early Pleistocene hominins innovative abilities have been underplayed in the Nihewan Basin, and in Eastern Asia, more broadly. While the Nihewan assemblages are often typed as the part of a "small lithic artefact tradition" [64, 65], such classifications hide some important technological variability, indicating that Nihewan assemblages are not homogeneous and unchanging across their long duration, i.e., from 1.7 Ma to 1 Ma.

The question arises as to why there appears to be increasing technological innovations at ca. 1.2–1.1 Ma in the Nihewan. Though no clear explanation can be given, palaeoenvironmental evidence indicates that the DGT occupations generally correspond with the onset of the mid-Pleistocene climate transition, occurring at ca. 1.25–0.8 Ma [34, 35, 37]. In high-latitude areas of North China, sediment grain sizes, rock magnetic and pollen data record significant environmental fluctuations [36, 44–46]. Hence, compared to earlier occupations in the Nihewan, such as at Majuangou and Xiaochangliang, the DGT inhabitants likely faced more unstable environments, perhaps requiring novel adaptations, and thus leading to new technological innovations. Dennell [66] argued that the Nihewan Basin was not inhabitable during glacials and on a year-round basis in the Early Pleistocene owing to cold winters and highly seasonal environments. With the onset of the MPT, and more variable and colder environments, it is possible that Nihewan hominins were forced to innovate their toolkits even further, though palaeoenvironmental and seasonal data are sorely needed to test this hypothesis.

Conclusion

Here we provided the most up-to-date information on the DGT lithic assemblages, one of the richest Early Pleistocene sites in the Nihewan Basin of China. Lithic comparisons between XCL and DGT, two systematically studied assemblages, indicate that hominins in the Nihewan Basin, between 1.4–1.1 Ma, displayed considerable technological flexibility, utilizing both free-hand and bipolar techniques in variable frequency. In both of these cases, the Nihewan hominins were able to overcome limitations of small clast size and poor-quality materials in order to obtain sharp-edged implements. While utilizing the same types of raw materials, the DGT hominins demonstrate some significant changes in lithic reduction methods in comparison to XCL, including the application of more control and preparation in conchoidal flaking methods, resulting in efficient utilization of clasts and predetermined plans for the size and shape of the struck pieces. In addition, though XCL and DGT hominins retouched flakes in various

ways to produce specific tool forms, the frequency of retouched pieces at DGT was greater, with the production of rare tool types, such as borers and points. The production of frequent and diverse tool forms at DGT signals innovations in tool production and new activity tasks at 1.1 Ma, perhaps as a consequence of adaptations to more variable environments in the high latitudes during the MPT.

Though Early Pleistocene lithic assemblages in China are often grouped as Mode 1 or as part of a "simple core-flake technology" [30, 67], increasing variability is evident in reduction systems as archaeologists more closely examine early sites, both from a temporal and geographical perspective [15, 27, 58]. Evidence for core rotation and bifacial working of small clasts in Early Pleistocene industries indicate that hominins in East Asia had the potential ability to fashion bifacial implements and large cutting tools [68–70] when the opportunity was presented in these somewhat younger lithic assemblages. This would imply that, in some cases, hominins present in Eastern Asia were the potential makers of Acheulean-like tools, without necessarily requiring a dispersal of Acheulean hominins from elsewhere.

Acknowledgments

We thank Xiao-Cong GUO of the Institute of Vertebrate Paleontology and Paleoanthropology, Chinese Academy of Sciences, for helping prepare Fig 5. MDP wishes to acknowledge the support of the Max Planck Society. We thank the Editor, Robin Dennell and an anonymous reviewer for their assistance and insightful comments, which helped us to improve this article.

Author Contributions

Data curation: Shi-Xia Yang, Jian-Ping Yue.

Formal analysis: Jian-Ping Yue.

Funding acquisition: Shi-Xia Yang, Cheng-Long Deng, Ri-Xiang Zhu.

Project administration: Shi-Xia Yang, Michael D. Petraglia, Ya-Mei Hou, Ri-Xiang Zhu.

Resources: Ya-Mei Hou.

Writing – original draft: Shi-Xia Yang, Michael D. Petraglia.

Writing – review & editing: Shi-Xia Yang, Michael D. Petraglia, Cheng-Long Deng.

References

1. Movius HL. The Lower Paleolithic cultures of southern and eastern Asia. T Am Philos Soc. 1948; 38(4): 329–420.
2. Ikawa-Smith F. Introduction: The Early Paleolithic tradition of East Asia. In: Ikawa-Smith F, editor. Early Paleolithic in South and East Asia. The Hague: Mouton Publishers; 1978. pp. 1–10.
3. Gao X, Norton CJ. A critique of the Chinese 'Middle Palaeolithic'. Antiquity. 2002; 76(292): 397–412.
4. Leakey MD. Olduvai Gorge: Excavations in Beds I and II, 1960–1963. Cambridge: Cambridge University Press; 1971.
5. Semaw S, Rogers M, Stout D. The Oldowan-Acheulian transition: Is there a 'developed Oldowan' artifact tradition? In: Camps M, Chauhan PR, editors. Sourcebook of Paleolithic Transitions. New York: Springer; 2009. pp. 173–193.
6. Texier PJ. The Oldowan assemblage from NY18 site at Nyabusosi (Toro-Uganda). C R Acad Sci Paris. 1995; 320: 647–653.
7. Burdukiewicz JM. Lower Palaeolithic transitions in the northern latitudes of Eurasia. In: Camps M, Chauhan PR, editors. Sourcebook of Paleolithic Transitions. New York: Springer; 2009. pp. 195–209.
8. Delagnes A, Roche H. Late Pliocene hominid knapping skills: The case of Lokalalei 2C, West Turkana, Kenya. J Hum Evol. 2005; 48(5): 435–472. https://doi.org/10.1016/j.jhevol.2004.12.005 PMID: 15857650

9. de la Torre I. Omo revisited: Evaluating the technological skills of Pliocene hominids. Curr Anthropol. 2004; 45: 439–465.
10. Wei Q, Meng H, Cheng S Q. New Paleolithic site from the Nihewan beds. Acta Anthropol Sin. 1985; 4 (3), 223–232 (In Chinese).
11. Schick KD, Toth N, Wei Q, Clark JD, Etler D. Archaeological perspectives in the Nihewan basin, China. J Hum Evol. 1991; 21(1): 13–26.
12. Hou YM. Naming and preliminary study on the category of the 'Donggutuo core'. Acta Anthropol Sin. 2003; 22: 279–291.
13. Liu Y, Hou YM, Ao H. Analysis of lithic technology of Lower Pleistocene sites and environmental information in the Nihewan Basin, North China. Quatern Int. 2013; 295: 215–222.
14. Wei Q. New observations on stone artifacts from the Donggutuo site. Acta Anthropol Sin. 2014; 33(3): 254–269.
15. Yang SX, Hou YM, Yue JP, Petraglia MD, Deng CL, Zhu RX. The lithic assemblages of Xiaochangliang, Nihewan Basin: Implications for Early Pleistocene hominin behaviour in North China. PLoS ONE. 2016; 11(5): e0155793. https://doi.org/10.1371/journal.pone.0155793 PMID: 27205881
16. Xie F, Li J, Liu LQ. Paleolithic Archeology in the Nihewan Basin. Shijiazhuang: Huashan Literature & Arts Press; 2006. pp. 278.
17. Zhu RX, Deng CL, Pan YX. Magnetochronology of the fluviolacustrine sequences in the Nihewan basin and implications for early human colonization of Northeast Asia. Quat Sci. 2007; 27: 922–944.
18. Deng CL, Zhu RX, Zhang R, Ao H, Pan YX. Timing of the Nihewan formation and faunas. Quaternary Res. 2008; 69: 77–90.
19. Barbour GB. The deposits of the Sankanho Valley. Bulletin of Geological Society of China. 1925; 4: 53–55.
20. Wang HQ, Deng CL, Zhu RX, Wei Q, Hou YM, Boëda E. Magnetostratigraphic dating of the Donggutuo and Maliang Paleolithic sites in the Nihewan Basin, North China. Quaternary Res. 2005; 64: 1–11.
21. Wang HQ. Magnetic properties of lacustrine sediments at the Donggutuo section in the Nihewan Basin and their environmental significance. Quat Sci. 2007; 27: 1081–1091.
22. Yuan BY, Xia ZK, Niu PS. Nihewan Rift and Early Man. Beijing: Geology Publishing House; 2011.
23. Hou YM. The 'Donggutuo core' from Donggutuo industry of Lower Pleistocene in the Nihewan basin, North China and its indication. Anthropologie. 2008; 112(3): 457–471.
24. Pei SW, Li XL, Liu DC, Ma N, Peng F. Preliminary study on the living environment of hominids at the Donggutuo site, Nihewan Basin. Chinese Sci Bull. 2009; 54(21): 3896–3904.
25. Wei Q, Li J, Pei SW. Palaeolithic sites and the ancient culture. In: Yuan BY, Xia ZK, Niu PS, editors. Nihewan Rift and Early Man. Beijing: Geological Publishing House; 2011. pp. 132–207.
26. Hou YM, Wei Q, Feng XW, Lin SL. Re-excavation at Donggutuo in the Nihewan Basin, North China. Quat Sci. 1999; (2): 139–147.
27. Hou YM. Donggutuo Lithic Industry of the Nihewan Basin, North China. PhD dissertation of Institute of Vertebrate Paleontology and Paleoanthropology, Chinese Academy of Sciences; 2000. pp. 1–112.
28. Chen C. The Early Pleistocene Lithic Assemblage and Human Behaviors in Nihewan Basin. Shanghai: Xuelin Press; 2003. pp. 422–447.
29. Li HM, Wang JD. Magnetostratigraphic study of several typical geologic sections in North China. In: Liu TS, editor. Quaternary Geology and Environment of China. Beijing: Ocean Press; 1982. pp. 33–38.
30. Schick KD, Zhuan D. Early Paleolithic of China and eastern Asia. Evol Anthropol. 1993; 2: 22–35.
31. Li HM, Yang XQ, Friedrich H, Li HT. High resolution magnetostratigraphy and deposition cycles in the Nihewan Basin (North China) and their significance for stone artifact dating. Quaternary Res. 2008; 69: 250–262.
32. Singer BS, Hoffman KA, Chauvin A, Coe RS, Pringle MS. Dating transitionally magnetized lavas of the late Matuyama Chron: Toward a new $^{40}Ar/^{39}Ar$ timescale of reversals and events. J Geophys Res. 1999; 104: 679–693.
33. Hilgen FJ, Lourens LJ, Van Dam JA. The Neogene period. In: Gradstein FM, Ogg JG, Schmitz MD, Ogg GM, editors. The Geologic Time Scale 2012. Amsterdam: Elsevier BV; 2012. pp. 923–978.
34. Clark PU, Archer D, Pollard D, Blum JD, Rial JA, Brovkin V, et al. The Middle Pleistocene transition: Characteristics, mechanisms, and implications for long-term changes in atmospheric pCO2. Quaternary Sci Rev. 2006; 25: 3150–3184.
35. Mudelsee M, Schulz M. The Mid-Pleistocene climate transition: Onset of 100 ka cycle lags ice volume build-up by 280 ka. Earth Planet Sci Lett. 1997; 151(1): 117–123.

36. Ding ZL, Derbyshire E, Yang SL, Yu ZW, Xiong SF Liu TS. Stacked 2.6-Ma grain size record from the Chinese loess based on five sections and correlation with the deep-sea $\delta^{18}O$ record. Paleoceanography. 2002; 17(3): 1033.
37. Medina-Elizalde M, Lea DW. The Mid-Pleistocene transition in the tropical Pacific, Science. 2005; 310: 1009–1012. https://doi.org/10.1126/science.1115933 PMID: 16223985
38. Zhu RX, Hoffman KA, Potts R, Deng CL, Pan YX, Guo B, et al. Earliest presence of humans in northeast Asia. Nature. 2001; 413: 413–417. https://doi.org/10.1038/35096551 PMID: 11574886
39. Zhu RX, Potts R, Xie F, Hoffman KA, Deng CL, Shi CD, et al. New evidence on the earliest human presence at high northern latitudes in northeast Asia. Nature. 2004; 431: 559–562. https://doi.org/10.1038/nature02829 PMID: 15457258
40. Li YC, Xu QH, Yang XL. Pollen records of the Feiliang Paleolithic site in Yangyuan, Hebei Province. Geogr Territ Res. 1996; 12(3): 55–59.
41. Yuan BY, Zhu RX, Tian WL, Cui JX, Li RQ, Wang Q, et al. The age, subdivision and correlation of Nihewan Group. Sci China Ser D. 1996; 26(1): 67–73.
42. Xu QH, Wang ZH, Yang XL. Guess the climate and environment of the ancient life in Cenjiawan Paleolithic site based on the sporo-pollen analyze. In: Hebei Province Institute of Cultural Relics, editor. Archaeological Corpus of Hebei. Beijing: Oriental Press; 1998. pp. 505–513.
43. Ding ZL, Derbyshire E, Yang SL, Sun JM, Liu TS. Stepwise expansion of desert environment across northern China in the past 3.5 Ma and implications for monsoon evolution, Earth Planet. Sci. Lett. 2005; 237: 45–55.
44. An ZS, Huang YS, Liu WG, Guo ZT, Clemens S, Li L, et al. (2005), Multiple expansions of C4 plant biomass in East Asia since 7 Ma coupled with strengthened monsoon circulation. Geology. 2005; 33: 705–708.
45. Deng CL, Shaw J, Liu QS, Pan YX, Zhu RX. Mineral magnetic variation of the Jingbian loess/paleosol sequence in the northern Loess Plateau of China: Implications for Quaternary development of Asian aridification and cooling. Earth Planet Sci Lett. 2006; 241(1–2): 248–259.
46. Wu FL, Fang XM, Ma YZ, Herrmann M, Mosbrugger V, An ZS, et al. Plio-Quaternary stepwise drying of Asia: Evidence from a 3-Ma pollen record from the Chinese Loess Plateau. Earth Planet Sci Lett. 2007; 257(1–2): 160–169.
47. Raymo ME, Oppo DW, Curry W. The mid-Pleistocene climate transition: A deep sea carbon isotopic perspective. Paleoceanography. 1997; 12: 546–559.
48. Pei SW, Hou YM. Preliminary study on raw materials exploitation at Donggutuo site, Nihewan Basin, North China. Acta Anthropol Sin. 2001; 21: 271–281.
49. Shen C, Gao X, Wei Q. The earliest hominin occupations in the Nihewan Basin of northern China: Recent progress in field investigations. In: Norton CJ, Braun DR, editors. Asian Paleoanthropology. New York: Springer; 2011. pp. 169–180.
50. Diez-Martín F, Yustos PS, Domínguez-Rodrigo M, Prendergast ME. An experimental study of bipolar and freehand knapping of Naibor Soit quartz from Olduvai Gorge (Tanzania). Am Antiquity. 2011; 76 (4): 690–708.
51. Gurtov AN, Eren MI. Lower Paleolithic bipolar reduction and hominin selection of quartz at Olduvai Gorge, Tanzania: What's the connection? Quatern Int. 2014; 322: 285–291.
52. Byrne F, Proffitt T, Arroyo A, de la Torre I. A comparative analysis of bipolar and freehand experimental knapping products from Olduvai Gorge, Tanzania. Quatern Int. 2015. https://doi.org/10.1016/j.quaint.2015.08.018
53. de la Peña P. A qualitative guide to recognize bipolar knapping for flint and quartz. Lithic Technology. 2015; 40(4): 316–331.
54. Toth NP. The Stone Technologies of Early Hominids at Koobi Fora, Kenya: An Experimental Approach. PhD dissertation of University of California, Berkeley; 1982.
55. Inizan ML, Reduron-Ballinger M, Roche H, Tixier J. Technology and Terminology of Knapped Stone. Nanterre: Cercle de Recherches et d'Etudes Prehistoriques; 1999. pp. 96–100.
56. Pelegrin J. Remarks about archaeological techniques and methods of knapping: Elements of a cognitive approach to stone knapping. In: Roux V, Bril B, editors. Stone Knapping: The Necessary Condition for a Uniquely Hominid Behavior. Cambridge: McDonald Institute for Archaeological Research; 2005. pp. 23–33.
57. Chen C, Shen C, Chen WY. Lithic analysis of the Xiaochangliang industry. Acta Anthropol Sin. 2002; 21(1): 23–40.
58. Shen C, Wei Q. Lithic technological variability of the Middle Pleistocene in the eastern Nihewan Basin, northern China. Asian Perspect. 2004; 43(2): 281–301.

59. Wang HQ, Deng CL, Zhu RX, Xie F. Paleomagnetic dating of the Cenjiawan Paleolithic site in the Nihewan Basin, northern China. Sci China Ser D. 2006; 49: 295–303.
60. Xie F, Cheng SQ. Paleolths excavation in Cenjiawan village, Yangyuan county, Hebei province. Acta Anthropol Sin. 1990; 9: 265–272.
61. Xie F, Li J. The application of lithic refitting method in the research of Cenjiawan site. Journal of Chinese Antiquity. 1995; (7): 25–38
62. Keates SG. Evidence for the earliest Pleistocene hominid activity in the Nihewan Basin of northern China. Quatern Int. 2010; 223: 408–417.
63. Guan Y, Wang FG, Xie F, Pei SW, Zhou ZY, Gao X. Flint knapping strategies at Cenjiawan, an Early Paleolithic site in the Nihewan Basin, North China. Quatern Int. 2016; 400: 86–92.
64. Jia LP, Gai P, You YZ. The report of excavation at Shiyu site in Shanxi province. Acta Archaeological Sinica. 1972; 1: 39–58.
65. Du SS, Liu FL. The cause analysis of the small lithic artefacts of North China. In: School of Archaeology and Museology of Peking University, editor. A Collection of Studies on Archaeology 7. Beijing: Science Press; 2008. pp. 232–243.
66. Schick KD. A comparative perspective on Paleolithic cultural patterns. In: Akazawa T, Aoki K, Bar-Yosef O, editors. Neandertals and Modern Humans in Western Asia. New York: Plenum Press; 1998. pp. 449–460.
67. Dennell RW. The Nihewan Basin of North China in the Early Pleistocene: Continuous and flourishing, or discontinuous, infrequent and ephemeral occupation. Quatern Int. 2013; 295(438):223–236.
68. Hou YM, Potts R, Yuan BY, Guo ZT, Deino A, Wang W, et al. Mid-Pleistocene Acheulean-like stone technology of the Bose basin, South China. Science. 2000; 287(5458): 1622–1626. PMID: 10698732
69. Kuman K, Li H, Li C. Large cutting tools from the Danjiangkou Reservoir Region, central China: Comparisons and contrasts with western and South Asian Acheulean. Quatern Int. 2015; 400: 58–64.
70. Li H, Kuman K, Li CR. What is currently (un) known about the Chinese Acheulean, with implications for hypotheses on the earlier dispersal of hominids. C R Palevol. 2016. https://doi.org/10.1016/j.crpv.2015.09.008
